# Supplementary material for: Gap-state engineering of visible-light-active ferroelectrics for photovoltaic applications
Source: Nat Commun. 2017 Aug 8;8:207. doi: 10.1038/s41467-017-00245-9 (PMC5547143; doi:10.1038/s41467-017-00245-9)
Supplement: Supplementary file 1 — Supplementary Information [file 41467_2017_245_MOESM1_ESM.pdf]

File Name: Supplementary Information
Description: Supplementary Figures, Supplementary Tables, Supplementary Notes and Supplementary References

File Name: Peer Review File
Description:

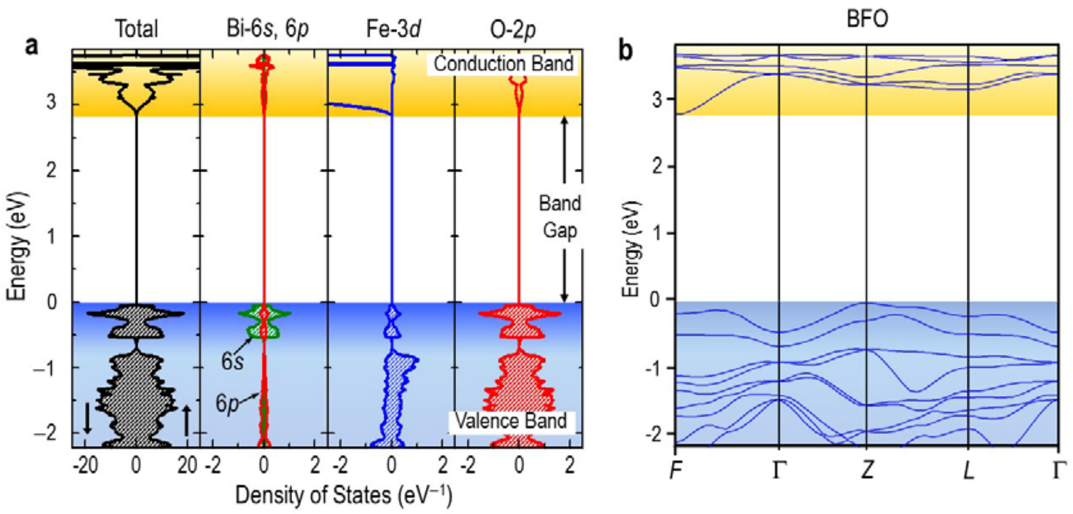

Supplementary Figure 1. Electronic structure of BFO calculated by the density functional MBJLDA (U–J = 4 eV for Fe-*d*): (**a**) total DOS and PDOS of Bi-6*s* and -6*p*, Fe-3*d* and O-2*p*; (**b**) electronic band structure. The results show that BFO has a band gap of 2.8 eV, which is in good agreement with experiments (2.7 eV[1,2]) and the value obtained by the screened exchange (sX) method reported in ref. 3 (2.8 eV). Our calculations indicate that BFO exhibits an indirect band gap with the VBM at the *Z* point and the CBM at the *F* point, where the position of the VBM is slightly different from that of the sX calculations[3].

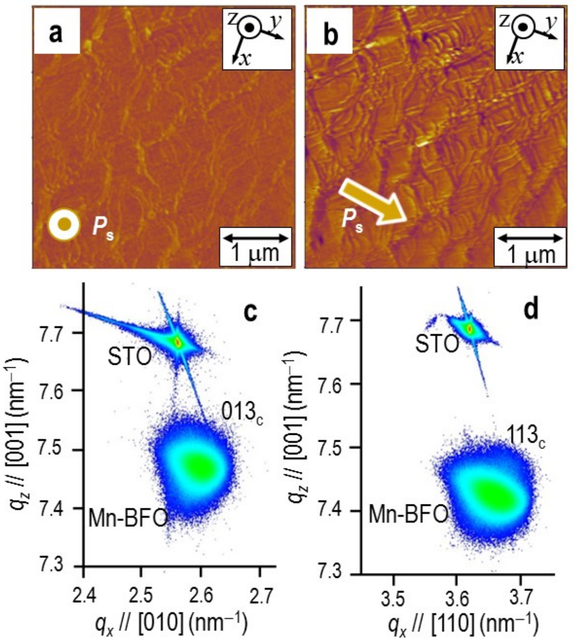

Supplementary Figure 2. Crystal and domain structures of the Mn-BFO thin film. Piezoresponse force microscopy (PFM) images (phase) observed in the (**a**) out-of-plane (OOP) and (**b**) in-plane (IP) modes. Both the OOP and the IP images exhibit uniform contrast, demonstrating that the Mn-BFO film has a single-domain (SD) structure with P_s in the [111] direction. X-ray diffraction reciprocal space maps (RSMs) of the (**c**) 013 and (**d**) 113 reflections of the Mn-BFO film. We did not find any split of the diffraction spots, which is consistent with the PFM result indicating the SD structure. In (**a**) and (**b**), the components of the P_s vector are denoted by a doted circle or arrow. We confirmed that the BFO film is in the SD state, as reported in ref. 4.

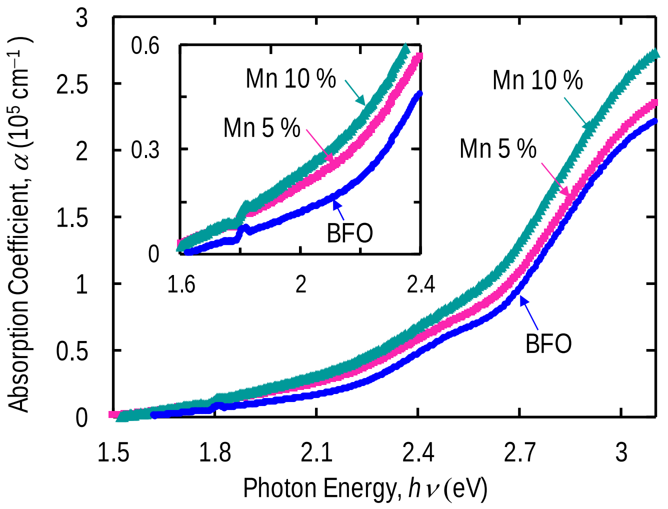

Supplementary Figure 3. Absorption spectra observed for the BFO and Mn-BFO (Mn 5 % and 10 %) films; absorption coefficient (α) as a function of photon energy ($h\nu$). The BFO film has a strong absorption at ~3 eV derived from a *dipole-allowed p-d* charge transfer (CT) transition and an absorption at ~2.5 eV, which is assigned to a *dipole-forbidden p-d* CT transition[5]. An apparent absorption at ~2 eV was also observed, which is tentatively attributed to the unoccupied gap (b_1) state arising from Fe^{4+} adjacent to oxygen vacancies as a result of the formation of Bi vacancies[6]. The apparent absorption at ~2.5 eV with a tail has been also reported in refs. 5, 7, 8. We note that the Mn doping enhances α under illumination with below-bandgap light in addition to above-bandgap light. The α at 2 eV of the Mn-BFO (5 %) film is 1.9×10^4 cm^{-1}, which is approximately twice that of the BFO film. These results show that the half-filled gap state given by the Mn doping increases α in a broad $h\nu$ range. The irregular change in α at around 1.8 eV is due to the switching of diffraction gratings in our ultraviolet-visible (UV-Vis) spectrometer.

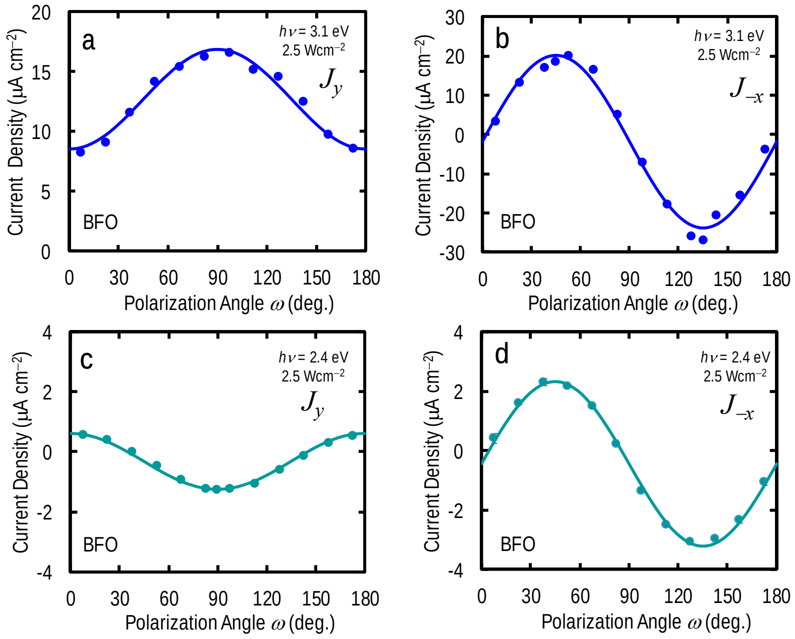

Supplementary Figure 4. Light polarization angle ω dependences of short-circuit currents of the BFO film: (**a**) J_y and (**b**) J_{-x} at $h\nu = 3.1$ eV; (**c**) J_y and (**d**) J_{-x} at $h\nu = 2.4$ eV. A sinusoidal dependence on ω with an offset is seen for J_y, while J_{-x} shows only the sinusoidal oscillation. We cannot detect the PV response under illumination at $h\nu$ = 1.9 eV.

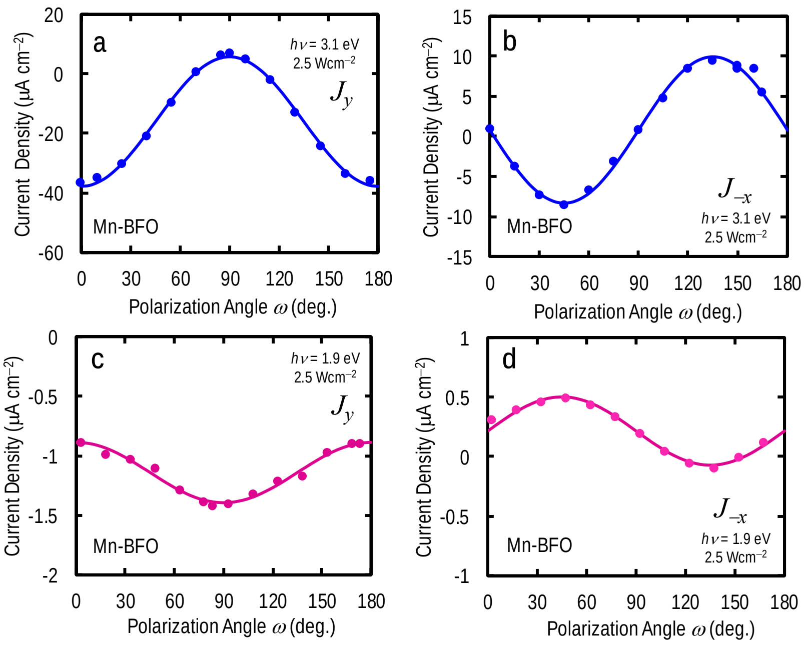

Supplementary Figure 5. Light polarization angle ω dependences of short-circuit currents of the Mn-BFO (5 %) film: (**a**) J_y and (**b**) J_{-x} at $h\nu = 3.1$ eV; (**c**) J_y and (**d**) J_{-x} at $h\nu = 1.9$ eV. The film shows the sinusoidal dependences on ω of J_y and J_{-x} even at $h\nu = 1.9$ eV.

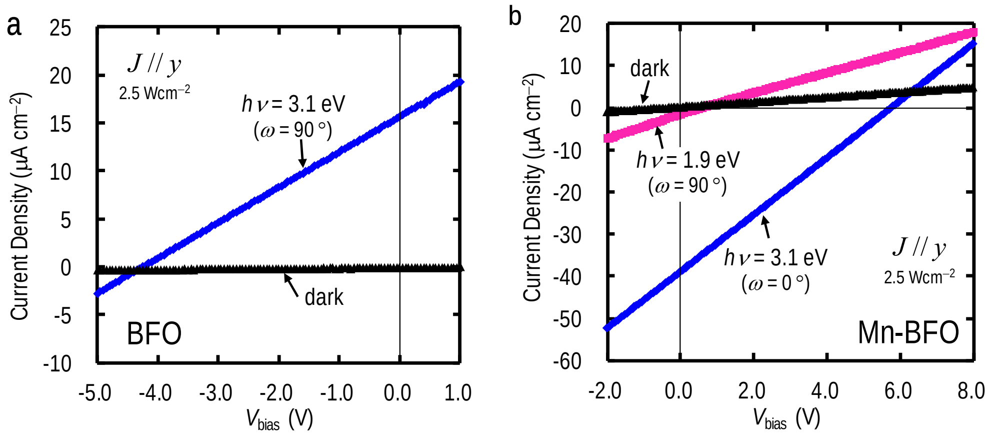

Supplementary Figure 6. Current density-bias voltage (J-V_{bias}) properties along the y direction of the films of (**a**) BFO under dark and illumination at $h\nu$ =3.1 eV and (**b**) Mn-BFO (5 %) under dark and illumination at $h\nu$= 3.1 eV and 1.9 eV. The polarization angle ω is aligned so that the PV response becomes maximum in each respective measurement (the values are show in parentheses).

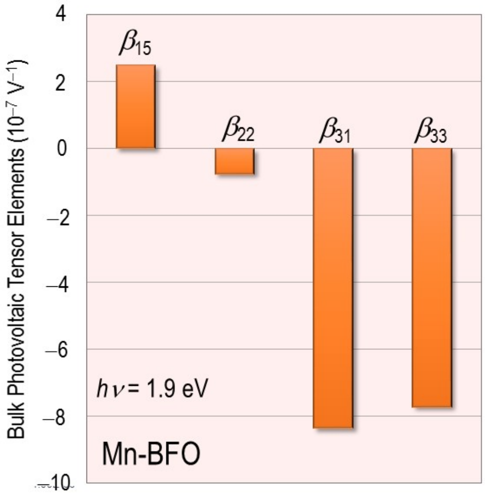

Supplementary Figure 7. Bulk photovoltaic (BPV) tensor elements (β_{15}, β_{22}, β_{31} and β_{33}) of the Mn-BFO (5 %) film under illumination at $h\nu$ = 1.9 eV. Those of the BFO film cannot be obtained owning to a negligible photoresponse at $h\nu$ = 1.9 eV.

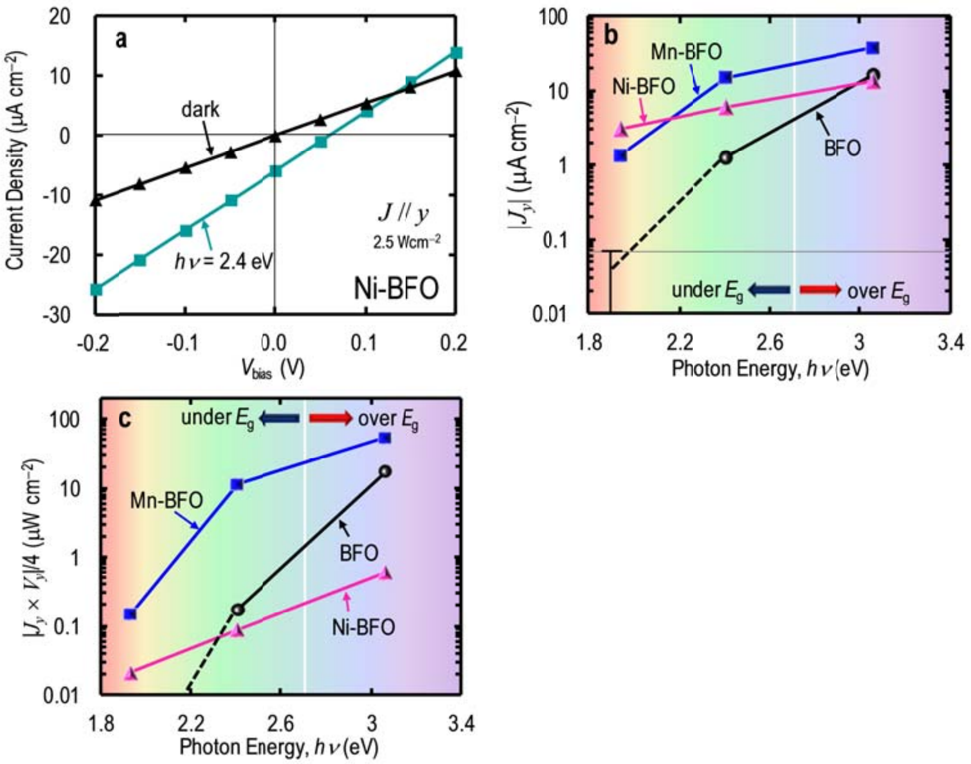

Supplementary Figure 8. (**a**) J-V_{bias} properties along the y direction of the Ni (5 %)-doped BFO (Ni-BFO) film in the single-domain state under dark and illumination at $h\nu = 2.4$ eV. The film was annealed in an O_3 atmosphere ($P_{O3} = 1$ Pa) at 350 °C for 1 h (the details are described in **Supplementary Note 3**). Photon energy ($h\nu$) dependences of (**b**) $|J_y|$ and (**c**) $|J_y \times V_y|/4$, where $|J_y \times V_y|/4$ represents photogenerated power per unit cross-sectional area. The horizontal line in (**b**) denotes the detection limit in our measurements. For the Ni-BFO film at $h\nu = 2.4$ eV, an apparent $|J_y|$ appears whereas $|V_y|$ is 0.06 V. Even though the $|J_y|$ at $h\nu < E_g$ is substantially large compared with the BFO film, the $|V_y|$ is as small as ~ 0.1 V and then the $|J_y \times V_y|/4$ becomes lower.

Considering the electronic structure of Ni-BFO in which the unoccupied gap state exists near the VBM (over 2.5 eV lower in energy with respect to the CBM, see **Fig. 1**), illumination with $h\nu$ of 1.9 eV and 2.4 eV is not capable of generating an electron-hole pair and can pump electron only from the VB to the gap state. This one-photon process leaves behind a photogenerated hole, thereby increasing $|J_y|$ at $h\nu < E_g$ by the Ni doping.

We note that a further enhancement in $|J_y|$ is achieved at $h\nu = 2.4$ eV by the Mn doping and that the Mn-BFO film outputs a $|J_y \times V_y|/4$ by two orders of magnitude higher. These results lead to the conclusion that the two-photon process via the half-filled gap states is more effective in the BPV effect compared to the one-photon process.

Supplementary Table 1. BPV tensor elements β_{ij} of the BFO and Mn-BFO (5 %) films at $h\nu$ of (**a**) 3.1 eV, (**b**) 2.4 eV and (**c**) 1.9 eV.

(**a**) $h\nu$= 3.1 eV

	β_{15} (V^{-1})	β_{22} (V^{-1})	β_{31} (V^{-1})	β_{33} (V^{-1})
BFO	8.1×10^{-5}	-1.1×10^{-5}	6.4×10^{-5}	-1.1×10^{-4}
Mn-BFO	-1.1×10^{-5}	-2.4×10^{-5}	4.0×10^{-5}	-2.4×10^{-4}

(**b**) $h\nu$= 2.4 eV

	β_{15} (V^{-1})	β_{22} (V^{-1})	β_{31} (V^{-1})	β_{33} (V^{-1})
BFO	3.0×10^{-6}	-4.4×10^{-7}	1.6×10^{-6}	-4.4×10^{-6}
Mn-BFO	7.2×10^{-6}	-1.4×10^{-6}	-1.5×10^{-5}	-1.4×10^{-5}

(**c**) $h\nu$= 1.9 eV

	β_{15} (V^{-1})	β_{22} (V^{-1})	β_{31} (V^{-1})	β_{33} (V^{-1})
BFO [a]	—	—	—	—
Mn-BFO	2.5×10^{-7}	-7.7×10^{-8}	-8.4×10^{-7}	-7.7×10^{-7}

[a] Photocurrents were below the detection limit.

Supplementary Table 2. Glass coefficients $G_{ij,k}$ at $h\nu$ of (**a**) 3.1 eV, (**b**) 2.4 eV and (**c**) 1.9 eV.

(**a**) $h\nu$= 3.1 eV

	$G_{15,k}$ (cmV^{-1})	$G_{22,k}$ (cmV^{-1})	$G_{31,k}$ (cmV^{-1})	$G_{33,k}$ (cmV^{-1})
$k = 1$ [a]	3.8×10^{-10}	-5.2×10^{-11}	3.0×10^{-10}	-5.2×10^{-10}
$k = 2$ [b]	-7.7×10^{-9}	-1.1×10^{-9}	-2.0×10^{-9}	-1.1×10^{-8}

(**b**) $h\nu$= 2.4 eV

	$G_{15,k}$ (cmV^{-1})	$G_{22,k}$ (cmV^{-1})	$G_{31,k}$ (cmV^{-1})	$G_{33,k}$ (cmV^{-1})
$k = 1$ [a]	6.3×10^{-11}	-9.2×10^{-12}	3.3×10^{-11}	-9.2×10^{-11}
$k = 2$ [b]	4.0×10^{-10}	-9.2×10^{-11}	-1.6×10^{-9}	-9.2×10^{-10}

(**c**) $h\nu$= 1.9 eV

	$G_{15,k}$ (cmV^{-1})	$G_{22,k}$ (cmV^{-1})	$G_{31,k}$ (cmV^{-1})	$G_{33,k}$ (cmV^{-1})
$k = 1$ [a, c]	—	—	—	—
$k = 2$ [b]	3.7×10^{-11}	-1.2×10^{-11}	-1.3×10^{-10}	-1.2×10^{-10}

[a] $G_{ij,1}$ represents the photogenerated strength arising from the one-photon optical transition of the VB–the CB.

[b] $G_{ij,2}$ reflects the photogenerated strength arising from the charge transfer transition via the Mn-3d-derived gap (2e) state.

[c] Photocurrents were below the detection limit.

Supplementary Table 3. Conductivities (σ) under dark and illumination.

	Conductivity σ (S cm^{-1})			
$h\nu$ (eV)	BFO	Mn-BFO	Ni-BFO [c] (annealed)	Ni^{2+}($V_O^{\bullet\bullet}$)-BFO [d] (as-deposited)
3.1	1.5×10^{-7}	2.6×10^{-7}	3.0×10^{-6}	1.6×10^{-7}
2.4	9.3×10^{-8}	1.9×10^{-7}	4.0×10^{-6}	1.1×10^{-7}
1.9	— [a]	1.2×10^{-7}	4.4×10^{-6}	6.4×10^{-8}
1.5	— [a]	3.6×10^{-8}	— [b]	— [b]
Dark	2.5×10^{-9}	2.4×10^{-8}	2.2×10^{-6}	2.9×10^{-9}

[a] Photocurrents were below the detection limit.

[b] Photocurrents were not measured.

[c] The annealing in an O_3 atmosphere leads to a high σ under dark by 2–3 orders of magnitude compared with the BFO and Mn-BFO films, showing that the valence state of Ni in the annealed film is expressed by $Ni^{2+}\underline{L}$, where $\underline{L}$ denotes a ligand hole (see main text).

[d] The as-deposited film has a valence state of Ni^{2+} with oxygen vacancy ($V_O^{\bullet\bullet}$), which is confirmed by their low σ values (its electronic structure is discussed in **Supplementary Note 3**).

Supplementary Note 1. DFT calculation method

Taking account of the magnetic structure of rhombohedral BFO, which can be approximated as a *G*-type antiferromagnetic ordering[9], we adopted the antiferromagnetic spin configuration formed by the *d* electrons of the Fe atoms. Prior to the structural optimization, either a magnetic moment of +5 μ_B (μ_B denotes the Bohr magneton) or −5 μ_B was put to each Fe atom to construct the antiferromagnetic ordering, which changes the space group from *R*3*c* to *R*3. The structural optimization of the BFO unit cell was performed with the Monkhorst–Pack *k*-point mesh of 6 × 6 × 6 centred on the Γ point in the Brillouin zone until the Hellmann–Feynman force on each atom was smaller than 0.1 eV/nm. The optimization calculation yielded a total magnetic moment of 0 μ_B.

We employed the supercell approach to perform the DFT calculations for TM-doped BFO, one of which (TM = Mn) is displayed in **Fig. 2(a)**. First, we formed a supercell of 80 atoms, i.e., 2 × 2 × 2, constructed from the primitive BFO cell. We replaced one of the Fe atoms with a negative magnetic moment by TM and then obtained the supercell of $Bi_{16}(Fe_{15}TM)O_{48}$ in rhombohedral *R*3 symmetry (TM = Cr, Mn, Co, Ni and Cu), which corresponds to an approximately 6 % TM doping. We set the magnetic moments of the TMs as −3 μ_B (Cr), −4 μ_B (Mn, Co), −1 μ_B (Ni) and −2 μ_B (Cu). The structural optimizations led to total magnetic moments of 2 μ_B (TM = Cr), 1 μ_B (TM = Mn), 3 μ_B (TM = Co), 3 μ_B (TM = Ni) and 3 μ_B (TM = Cu). The detailed result of TM = Ni (*S* = 1) is described in the main text.

Supplementary Note 2. Degeneracy of the 2*e* (gap) state of Mn-doped BFO

We carefully checked the possibility that the Jahn-Teller effect lifts the degeneracy of the 2*e* state. In general, the symmetry in space group *R*3 (point group C_{3v}) splits the *d*-derived orbitals into a_1, 1*e* and 2*e*, and the 2*e* state appears above the a_1 state. One exception has been reported for Fe-doped $LiNbO_3$, with the d^6 electron configuration of Fe, where the a_1 state (filled) is markedly stabilized with respect to the 1*e* and 2*e* states (unoccupied) in the minority-spin band[10,11]. In Mn-doped BFO with the d^4 electron configuration of Mn, if the local symmetry around Mn is broken by the Jahn-Teller effect, the degeneracy of the 2*e* states is lifted, and then the d_{yz}- and d_{zx}-derived orbitals have different energy levels.

We considered the following initial structures of the $Bi_{16}(Fe_{15}TM)O_{48}$ supercell in the triclinic *P*1 space group (without any symmetry constraints): one is that Mn inherits the same fractional coordinates of the original Fe atom that is replaced, and the other is that Mn is intentionally located away from the three-fold rotation axis so that the

original symmetry is broken completely. We found that the geometry optimization calculations converge to the rhombohedral *R*3 structure. These results show that the energy gain given by maintaining the symmetry is larger than the stabilization energy associated with a Jahn-Teller distortion of the MnO_6 octahedron. We therefore conclude that the ground state of Mn-doped BFO has a rhombohedral *R*3 structure.

Supplementary Note 3. Oxidation state of Ni in BFO before and after the O_3 annealing

To confirm the oxidation state of the Ni-doped film, we performed the annealing in an O_3 atmosphere (P_{O3} = 1 Pa) at 350 °C for 1 h. The annealed film exhibits a dark conductivity (σ_{dark}) of 2.2×10^{-6} Scm^{-1}, which is approximately 3 orders of magnitude higher than that of the as-deposited film (2.9×10^{-9} Scm^{-1}, see **Supplementary Table 3**).

Considering a relatively reduced atmosphere during the film deposition and the marked increase in σ_{dark} by the O_3 annealing, we think that the incorporation of Ni in the as-deposited film is accompanied by oxygen vacancy ($V_O^{\bullet\bullet}$) for charge compensation, as expressed by

$$2\mathrm{NiO} \xrightarrow{\mathrm{Fe_2O_3}} 2\mathrm{Ni}'_{\mathrm{Fe}} + 2\mathrm{O}_{\mathrm{O}}^{*} + V_{\mathrm{O}}^{\bullet\bullet}, \quad (1)$$

where $\mathrm{Ni}'_{\mathrm{Fe}}$ denotes Ni^{2+} on the Fe^{3+} site and $\mathrm{O}_{\mathrm{O}}^{*}$ is O^{2-} on the O site (the Kröger-Vink notation is adopted). The O_3 annealing results in an oxidation reaction expressed by

$$2\mathrm{Ni}'_{\mathrm{Fe}} + V_{\mathrm{O}}^{\bullet\bullet} + \frac{1}{2}\mathrm{O}_2 \rightarrow 2\mathrm{Ni}'_{\mathrm{Fe}} \cdot h^{\bullet} + \mathrm{O}_{\mathrm{O}}^{*}, \quad (2)$$

where $\mathrm{Ni}'_{\mathrm{Fe}} \cdot h^{\bullet}$ represents $\mathrm{Ni}^{2+}\underline{L}$ ($\underline{L}$ is a ligand hole).

To provide information on the electronic structure of BFO containing Ni^{2+} and $V_O^{\bullet\bullet}$, at first the supercell of $Bi_{16}Fe_{14}Ni_2O_{48}$ having Ni atoms with a distance of ~ 0.7 nm was constructed and the structure was geometrically optimized in hexagonal *R*3 space group, yielding a total magnetic moment of 0 μ_B. The structure of the optimized cell was transformed to the rhombohedral primitive cell with *P*1 symmetry (without any symmetry constraints). One of the oxygen atoms is removed to create $V_O^{\bullet\bullet}$, which results in a defective cell of $Bi_{16}Fe_{14}Ni_2O_{47}$ with the valence state of Ni^{2+}. The position of $V_O^{\bullet\bullet}$ was varied from the 1st nearest-neighbor (NN) to the 10th NN site with respect to the Ni atom with a negative magnetic moment to find a stable site of $V_O^{\bullet\bullet}$. The fractional coordinates of all atoms were optimized in the fixed lattice parameters. These optimization calculations were done with the constraint of a total magnetic moment of 0 μ_B, which can ensure the antiferromagnetic spin ordering.

We found that the cell with $V_O^{\bullet\bullet}$ on the 1st NN site exhibits the lowest energy by ~

0.3 eV. These results indicate that $V_O^{••}$ is stabilized in the vicinity of Ni^{2+} and that the trapping of $V_O^{••}$ by Ni^{2+} changes the local structure: the presence of $V_O^{••}$ is accompanied by its adjacent NiO_5 and FeO_5 pyramids. We regard this supercell as $Ni^{2+}(V_O^{••})$-BFO.

Supplementary Figure 9 displays the energy positions of the gap states in $Ni^{2+}(V_O^{••})$-BFO obtained by the GGA-PBEsol+U calculations. Because the presence of $V_O^{••}$ results in Ni^{2+} and Fe^{3+} in their distorted pyramids and thereby the degeneracy of their 3d states is lifted, these cations create an empty gap state in the majority and minority spin band. The energy levels of the gap states are estimated to be 1.8–2.0 eV with respect to the VBM. This electronic structure can explain the low σ_{dark} of the as-deposited film, where the defect structure is described by $Ni^{2+}(V_O^{••})$-BFO.

As shown in **Fig. 1**, the electronic structure of Ni-BFO (exhibiting $Ni^{2+}\underline{L}$) with an empty gap state in the vicinity of the VBM suggests that free holes are present owing to thermal activation even at room temperature. We think that Ni-BFO exhibits p-type conduction with a relatively high conductivity, which is in good agreement with the high σ_{dark} observed for the annealed film (**Supplementary Table 3**).

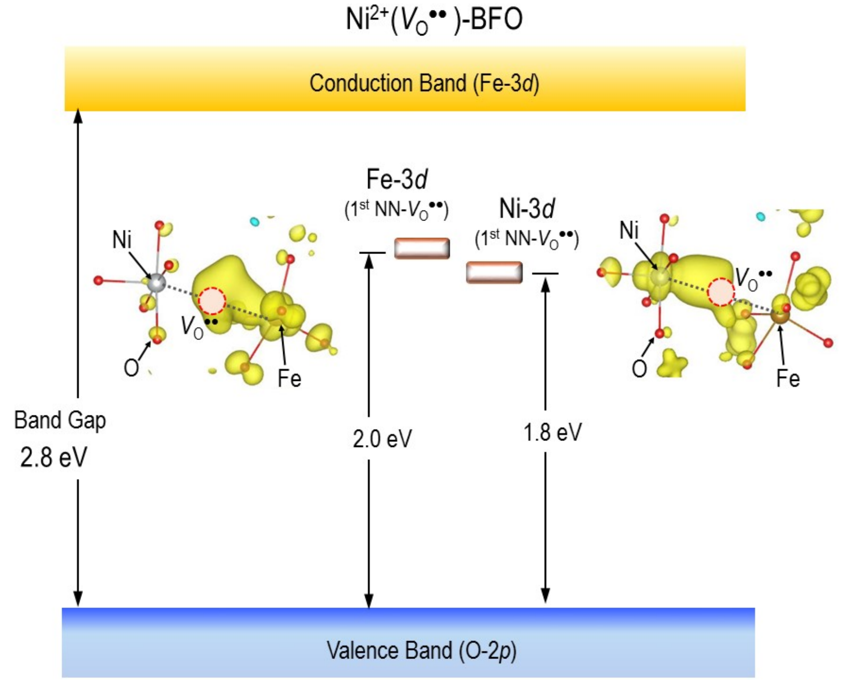

Supplementary Figure 9. Energy positions of the gap states in the $Ni^{2+}(V_O^{••})$-BFO cell ($Bi_{16}Fe_{14}Ni_2O_{47}$) with $V_O^{••}$ on the 1st-NN site obtained by the GGA-PBEsol+U calculations (U–J = 4 eV for Ni-3d and Fe-3d). Structural optimization calculations show that $V_O^{••}$ is stabilized in the vicinity of Ni^{2+}, yielding its adjacent NiO_5 and FeO_5. In these distorted pyramids, the degeneracy of the d orbitals is lifted, and thereby

creating an empty gap state in the majority-spin band at 1.8 eV (Ni-3*d* derived) and the minority-spin band at 2.0 eV (Fe-3*d* derived) with respect to the VBM. Because of a well-known GGA problem, the calculations underestimate the E_g (~ 2.3 eV), and the positions of the gap states are qualitative. For the sake of argument, we shift the CBM to 2.8 eV with respect to the VBM to describe the electronic structure of the host BFO lattice with E_g = 2.8 eV. This electronic structure with the empty gap states near the middle of the bandgap can explain the low σ_{dark} observed for the as-deposited Ni-doped film.

Supplementary Note 4. Estimation of absorption coefficient

Absorption coefficient α is evaluated as the following procedure, where multiple reflection is neglected because reflection at an interface between the ferroelectric film and the substrate is expected to be small (1 – 4 %), as deduced from the refractive indices reported in previous studies[12,13]. First, we evaluated transmittance (T_{STO}) and reflectance (R_{STO}) of the bare STO substrate. As schematized in **Supplementary Figure. 10**(**a**), the absorption of the substrate (A_{STO}) is expressed as $A_{\mathrm{STO}} = 1 - T_{\mathrm{STO}} - R_{\mathrm{STO}} = A'_{\mathrm{STO}}(1 - R_{\mathrm{STO}})$, where A'_{STO} represents the absorption by the substrate with respect to the intensity of light penetrating into its surface. Then, we measured the absorption of the BFO/STO and Mn-BFO/STO samples. The absorption of the sample (A_0) can be calculated by $A_0 = 1 - T_0 - R_0$, where T_0 and R_0 are experimentally measured transmittance and reflectance of the sample [**Supplementary Figure. 10**(**b**)]. Because both the film with absorption A_1 and the STO substrate with absorption A_2 contribute to the total absorption, A_0 is written as $A_0 = A_1 + A_2$.

We estimate α of the ferroelectric film from the experimentally obtained values of A'_{STO}, A_0, T_0 and R_0. The intensity of light penetrating into the sample through the surface is written as $1 - R_0$. We obtain $A_1 = (1 - R_0)[1 - \exp(-\alpha t)]$, where t is a thickness of the ferroelectric layer. As the relative intensity of light reaching the film/substrate interface is denoted by $1 - R_0 - A_1$, we find $A_2 = A'_{\mathrm{STO}}(1 - R_0 - A_1)$. As a result, T_0 is expressed as $T_0 = 1 - R_0 - A_1 - A_2 = 1 - R_0 - (1 - R_0)[1 - \exp(-\alpha t)] - A'_{\mathrm{STO}}(1 - R_0 - A_1)$, which yields the following relationship: $\alpha = -\frac{1}{t}\ln\frac{T_0}{(1-R_0)(1-A'_{\mathrm{STO}})}$.

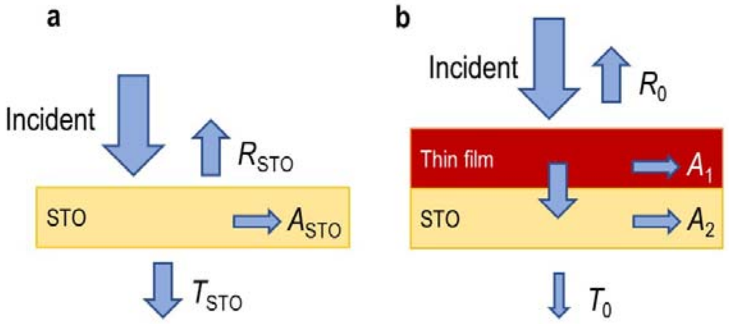

Supplementary Figure 10. Schematic illustration of light absorption and optical parameters for (**a**) the bare STO substrate and (**b**) the sample (the ferroelectric film and its substrate). Here, multiple reflection is neglected because reflection at an interface between the ferroelectric film and the substrate is as small as 1 – 4 % [12,13].

Supplementary Note 5. Optical transition process through the Mn-derived gap state

The optical transition via the Mn-derived gap state is accompanied by the change in the valence state of Mn. In the ground state of Mn-BFO with Mn^{3+}, electron pumping from the gap state to the CB turns Mn^{3+} to Mn^{4+}, while that from the VB to the gap state changes Mn^{3+} to Mn^{2+}. **Supplementary Figure 12** indicates the energy levels of the gap states derived from Mn^{4+}, Mn^{3+} and Mn^{2+} in BFO. The GGA-PBEsol+U calculations exhibit that the empty state of Mn^{4+} is positioned in the middle of the band gap while that of the filled state of Mn^{2+} is deep, where the difference of the CBM–the Mn^{2+} state is as high as 2.2 eV.

Here, we address the transitions induced by illumination with monochromatic light (hv = 1.5 eV, 1.9 eV, and 2.4 eV), where the optical processes involve lattice relaxations. Namely, an electron excitation occurs in the fixed ground-state structure, whereas the electronic excited state is followed by a lattice relaxation to attain a metastable state. In addition, when the excited state goes back to the ground state, an electron is captured in the fixed excited-state structure, and then the ground-state structure is established through a subsequent lattice relaxation. Here, we consider the majority carrier delivering the photoconduction by taking into account the results of photoconductivity (σ_{photo}) (**Supplementary Table 3**), and address the relations with the BPV effect.

5.1 *hv* = 1.5 eV

Only the optical transition of the VB–the Mn^{3+} state is induced under illumination with this photon energy. This involves the Mn^{3+}–Mn^{2+} change, leaving behind hole. Because the photon energy is smaller than the difference of the Mn^{2+} state–the CBM, the photogenerated hole is then captured by Mn^{2+} during its lifetime, and the Mn valence goes back to Mn^{3+}. We found that this illumination leads to a high σ_{photo} compared with the σ_{dark}, showing that the photogenerated hole is injected. In contrast, the short-circuit current density (J_{sc}) was below the detection limit (**Supplementary Figure 11**). A possible origin of the negligibly small J_{sc} is either a localization of the photoexcited hole around Mn^{2+} or its short carrier lifetime by the Shockley–Read–Hall (SRH) recombination owing to a small energy difference of the VBM–the Mn^{2+} state (~ 0.6 eV).

5.2 *hv* = 1.9 eV

This photon energy permits, in principle, the following two optical processes. One is a transition associated with the Mn^{3+}–Mn^{2+} change, after which the resultant hole is captured by the Mn^{2+} centre. This light cannot pump electron from the Mn^{2+} state to the CB, and therefore the photoexcited carrier is hole in this process (the same at $h\nu$ = 1.5 eV). The other is an optical transition of the Mn^{3+} state–the CB along with the Mn^{3+}–Mn^{4+} change. Because the difference of the VBM–the Mn^{4+} state is smaller than 1.9 eV, the Mn^{4+} state can receive electron from the VB by the photon absorption. This successive optical process results in an electron-hole pair. Considering the experimental fact that illumination at $h\nu$ = 1.5 eV induces no detectable PV response while increasing σ_{photo}, we think that the holes injected into the VB alone cannot explain the J_{sc} observed at $h\nu$ = 1.9 eV. Our experimental and DFT results suggest that the BPV effect at $h\nu$ = 1.9 eV stems from photogenerated electron-hole pairs.

5.3 *hν* = 2.4 eV

Under illumination at this photon energy, the optical processes triggered by both the Mn^{3+}–Mn^{2+} and Mn^{3+}– Mn^{4+} changes can generate electron hole pairs. In one process, the first transition of the VB–the Mn^{3+} state associated with the Mn^{3+}–Mn^{2+} change takes place, and afterwards the second transition of the Mn^{2+} state–the CB occurs. In the other process, the transition of the Mn^{3+} state–the CB along with the Mn^{3+}–Mn^{4+} change is followed by that of the VB–the Mn^{4+} state. These two kinds of optical processes caused by two-phonon absorption via the Mn^{3+} state leave an electron-hole pair. A high σ_{photo} observed at $h\nu$ = 2.4 eV compared at $h\nu$ = 1.9 eV features a high density of photogenerated electron-hole pair. Because two distinct pathways mediated through Mn^{2+} and Mn^{4+} contribute to the carrier generation, illumination at $h\nu$ = 2.4 eV can induce the robust BPV effect.

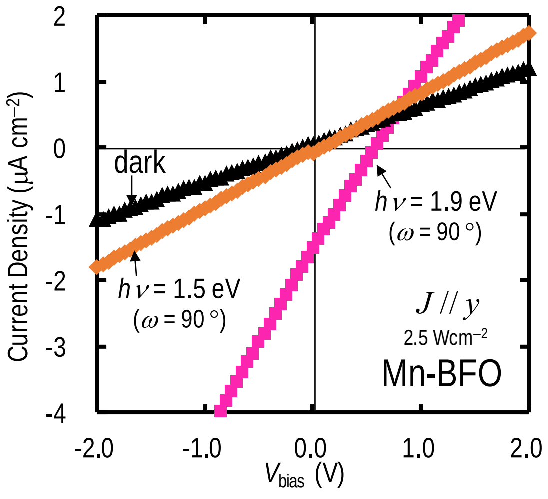

Supplementary Figure 11. J-V_{bias} properties along the y direction of the Mn-BFO (5 %) film under dark and illumination at $h\nu$ = 1.5 eV and 1.9 eV. An apparent photoresponse appears at $h\nu$ = 1.9 eV whereas that at $h\nu$ = 1.5 eV was below the detection limit.

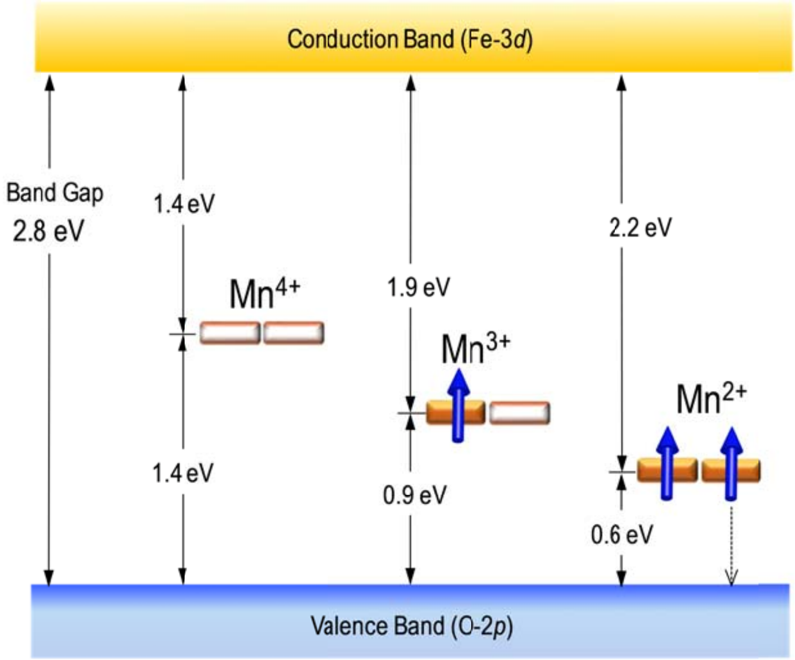

Supplementary Figure12. Energy levels of the gap states derived from Mn^{4+}, Mn^{3+}, and Mn^{2+} in BFO. The level of Mn^{3+} obtained by the MBJLDA calculations is shown. Because the MBJLDA method cannot be adopted for charged cells at present, those of Mn^{4+} and Mn^{2+} were calculated by the GGA-PBEsol+U method (U–J = 4 eV for Mn-3d and Fe-3d), where the background charge is controlled followed by the structural optimization of the fractional coordinates of the constituent atoms in the fixed unit-cell obtained for the Mn^{3+} cell. Although the GGA-PBEsol+U calculations underestimate the E_g (~ 2.3 eV), we ensure that the energy level of the Mn^{3+}-derived state is very close to that obtained by the MBJLDA calculations (the difference is ~ 0.05 eV). For the sake of argument, we shift the CBM to 2.8 eV with respect to the VBM to describe the electronic structure of the host BFO lattice with E_g = 2.8 eV.

Supplementary References

1. Ihlefeld, J. F. *et al.* Optical band gap of $BiFeO_3$ grown by molecular-beam epitaxy. *Appl. Phys. Lett.* **92,** 142908 (2008).
2. Basu, S. R. *et al.* Photoconductivity in $BiFeO_3$ thin films. *Appl. Phys. Lett.* **92,** 091905 (2008).
3. Clark, S. J. & Robertson, J. Band gap and Schottky barrier heights of multiferroic $BiFeO_3$. *Appl. Phys. Lett.* **90,** 132903 (2007).
4. Matsuo, H. *et al.* Bulk and domain-wall effects in ferroelectric photovoltaics. *Phys. Rev. B* **94,** 214111 (2016).
5. Pisarev, R. V, Moskvin, A. S., Kalashnikova, A. M. & Rasing, T. Charge transfer transitions in multiferroic $BiFeO_3$ and related ferrite insulators. *Phys. Rev. B* **79,** 235128 (2009).
6. Matsuo, H., Kitanaka, Y., Inoue, R., Noguchi, Y. & Miyayama, M. Switchable diode-effect mechanism in ferroelectric $BiFeO_3$ thin film capacitors. *J. Appl. Phys.* **118**, 114101 (2015).
7. Yamada, Y., Nakamura, T., Yasui, S., Funakubo, H. & Kanemitsu, Y. Measurement of transient photoabsorption and photocurrent of $BiFeO_3$ thin films: Evidence for long-lived trapped photocarriers. *Phys. Rev. B* **89**, 035133 (2014).
8. Xu, X. S. *et al.* Tunable band gap in $Bi(Fe_{1-x}Mn_x)O_3$ films. *Appl. Phys. Lett.* **96**, 192901 (2010).

9. Neaton, J. B., Ederer, C., Waghmare, U. V., Spaldin, N. A. & Rabe, K. M. First-principles study of spontaneous polarization in multiferroic $BiFeO_3$. *Phys. Rev. B* **71**, 014113 (2005).
10. Sanson, A. *et al.* Polaronic deformation at the Fe2+/3+ *impur*ity site in Fe:$LiNbO_3$ crystals impurity site in Fe:$LiNbO_3$ crystals. *Phys. Rev. B* **91**, 094109 (2015).
11. Noguchi, Y., Inoue, R. & Miyayama, M. Electronic origin of defect states in Fe-doped $LiNbO_3$ ferroelectrics. *Adv. Condens. Matter Phys*. **2016**, 2943173 (2016).
12. Kumar, A. *et al.* Linear and nonlinear optical properties of $BiFeO_3$. *Appl. Phys. Lett.* **92**, 121915 (2008).
13. Du, Y. *et al.* Optical properties of $SrTiO_3$ thin films by pulsed laser deposition. *Appl. Phys. A* **76**, 1105–1108 (2003).
